# Outcome Evaluation of a Multimodal Suicide Prevention Program Designed Through International Collaboration: Protocol for a Controlled Interrupted Time Series Study

**DOI:** 10.2196/66976

**Published:** 2025-09-19

**Authors:** Jean-Daniel Carrier, Marie-Claude Geoffroy, Michel Walter, Katia Dumont, Massimiliano Orri, Sofian Berrouiguet, Christophe Lemey, Monique Séguin

**Affiliations:** 1 Department of Psychiatry Faculty of Medicine and Health Sciences Université de Sherbrooke Sherbrooke, QC Canada; 2 McGill Group for Suicide Studies Douglas Mental Health University Institute McGill University Montreal, QC Canada; 3 Department of Psychiatry McGill University Montreal, QC Canada; 4 Mental Health Department Brest Medical University Hospital Brest France; 5 Centre de recherche de l'Hôpital Maisonneuve-Rosemont CIUSSS de l'Est-de-l'Île-de-Montréal Montreal, QC Canada; 6 School of Population and Global Health Department of Epidemiology, Biostatistics, and Occupational Health McGill University Montreal, QC Canada; 7 Danish Research Institute for Suicide Prevention Copenhagen Mental Health Centre Copenhagen Denmark; 8 Inserm UMR 1101, LATIM Brest France; 9 Department of Psychology Université du Québec en Outaouais Gatineau (Hull), QC Canada

**Keywords:** suicide, suicidal behaviors, prevention, multimodal program, complex intervention, outcome evaluation, interrupted time series

## Abstract

**Background:**

The Coopération Québec-France contre la Dépression et l’Isolement (CQFD-I) initiative represents a collaborative effort between Outaouais, a region in Quebec, and Brest, a metropolitan area in France, to reduce suicide through a multimodal approach. CQFD-I integrates 5 key strategies: a web portal on depression, education of primary care physicians on depression, training for mental health professionals in suicide risk assessment and management, and standardized postcrisis outreach and outpatient monitoring protocols.

**Objective:**

This paper aims to evaluate the effectiveness of CQFD-I in reducing outcomes related to suicidal behavior, namely suicide mortality, hospitalizations due to suicide attempts, and emergency room visits for suicide attempts, across the implementation sites compared to control areas.

**Methods:**

The evaluation will use a controlled, segmented interrupted time series design, spanning 3 distinct phases: preimplementation (2015 to 2020), implementation (2020 to 2022), and postimplementation (2022 to 2024) phases. The outcomes will be (1) suicide mortality, (2) hospitalizations for suicide attempts, and (3) emergency room visits for suicide attempts. Datasets covering September 2015 to August 2024 will be provided by public health organizations in both jurisdictions, that is, the Institut national de la santé publique du Québec and Santé publique France. In Quebec, control sites will consist of the 14 other health regions with a population of at least 50,000, while in France, control sites will include the 18 metropolitan areas with a status comparable to that of Brest Métropole. The analysis will focus on age- and sex-adjusted monthly rates of the specified outcomes, with an emphasis on changes across the study phases to assess the putative effectiveness of the CQFD-I intervention. We hypothesize that each study outcome will decrease by 20% at implementation sites compared to control sites between the preimplementation and postimplementation phases.

**Results:**

This project is funded by the Ministère de la Santé et des Services Sociaux du Québec, with access to data and expertise representing in-kind contributions from Santé publique France and Institut national de la santé publique du Québec. Data collection, led by the first author, will take place in September 2027 to allow for the delay required to confirm suicide deaths in official registers, with analysis planned between 2027 and 2028.

**Conclusions:**

To our knowledge, this study will be the first to document the outcomes of a multimodal suicide prevention program targeting geographically defined areas within 2 countries, not limited to the European Union.

**International Registered Report Identifier (IRRID):**

PRR1-10.2196/66976

## Introduction

### Background

Suicide, defined as the act of deliberately killing oneself, prematurely ends the lives of approximately 700,000 people every year around the globe [1,2]. For every death, it is estimated that there could be up to 20 suicide attempts [1]. A variety of effective strategies exist to counteract suicidal behaviors across diverse populations [3]. Best practices involve reaching as many people as possible by combining several strategies within a more comprehensive suicide prevention program [4], sometimes labeled as a multimodal, multilevel, multicomponent, community-based, or integrated intervention [5]. Multimodal programs are thought to be more effective than single strategies in preventing suicide deaths and attempts [6], especially programs implemented in high schools or targeting groups based on occupation or ethnicity [7,8]. However, current evidence is inconsistent regarding programs targeting the whole population of a geographic area [8].

Much of the available evidence on population-level multimodal suicide prevention pertains to a program named “Optimizing suicide prevention programs and their implementation in Europe” (OSPI-Europe), which provides community and professional training, public education, and direct intervention strategies to prevent suicide across the participating regions [9,10]. The first instance of OSPI-Europe implementation was the Nuremberg pilot in the early 2000s, a pre-post study with a control region that was underpowered to detect changes in suicide mortality but found a decrease of more than 30% in suicide attempts [11]. Building on the Nuremberg pilot and with support from the European Commission, OSPI-Europe was then implemented from 2008 to 2013 in 4 European regions [12], with additional instances of implementation in Germany [13-15] and Hungary [16].

Inspired by ongoing European trials, in 2014, government officials in France and the Canadian province of Quebec started working on an innovative suicide prevention project involving a shared vision of health and social services integration. The resulting Coopération Québec-France contre la Dépression et l’Isolement (CQFD-I) has since involved experts and financial contributions from both jurisdictions in designing and implementing a multimodal suicide prevention program in 2 demonstration sites: the Outaouais region in Quebec, Canada, and Brest Métropole in Brittany, France. The CQFD-I program was structured following the public health model, distinguishing 3 levels of preventive measures: universal, selective, and indicated strategies [17,18]. Interventions are classified as universal when they are relevant for the whole population, selective when they benefit at-risk populations, or indicated when they target people individually at risk. After navigating the lengthy process of designing a program compatible with both jurisdictions, and facing further delays caused by the COVID-19 pandemic, CQFD-I entered the postimplementation phase in September 2022.

OSPI-Europe evaluation studies compare implementation sites to areas chosen by stakeholders [11-13,15,16] or national suicide statistics [13-15]. To our knowledge, only 3 studies have evaluated the outcomes of whole population multimodal suicide prevention programs using formally matched controls, showing mixed results: a 7% suicide reduction in Australia [19]; no effect in New Zealand [20]; and mixed results from Japan’s NOCOMIT-J program, with notable decreases in rural areas [21]. Some studies incorporate time as a covariate [19-21] or use interrupted time series [22-24], which is crucial for understanding the sustained putative impact of such interventions and differentiating real changes from temporary fluctuations. However, evaluation studies of multimodal suicide prevention programs incorporating both a controlled group and interrupted time series in geographically specific programs are lacking.

### Objectives

In this paper, we present the protocol for the outcome evaluation of the CQFD-I program, the first instance of an international multimodal suicide prevention program with a non-European implementation. This study aims to determine whether implementing the CQFD-I multimodal suicide prevention program results in lower rates of suicidal behavior–related outcomes, that is, suicide mortality and suicide attempts, compared to matched control sites, and considering the effect of time.

## Methods

### Study Design

We will conduct an outcome evaluation of the CQFD-I program using a controlled, segmented interrupted time series design [25-27]. The 2 program implementation sites (the Outaouais region in Quebec, Canada, and Brest Métropole in Brittany, France) will serve as cases, each compared against a group of controls identified from geographic areas in the same jurisdictions that have not implemented the CQFD-I program [24]. Retrospective data will be sourced from national clinical administrative databases. This paper was written in accordance with the TREND (Transparent Reporting of Evaluations with Nonrandomized Designs) statement [28].

### Study Objective

Our objective is to assess whether implementing a multimodal suicide prevention program across 2 countries—encompassing a universal strategy (a web portal focused on depression and social isolation), selective strategies (training for primary care physicians on depression and training for mental health professionals on suicide risk assessment and management), and indicated strategies (standardized postcrisis outreach and outpatient monitoring practices)—leads to a reduction in outcomes associated with suicidal behavior, such as suicide mortality, hospitalization due to suicide attempts, and visits to emergency rooms (ERs) for suicide attempts, compared to control sites and over time. We hypothesize that after 2 years of the CQFD-I program’s implementation, there will be a 20% reduction in suicide mortality, hospitalizations due to suicide attempts, and ER visits for suicide attempts compared to control sites. This reduction will be observed when comparing the postimplementation phase (beginning in 2022) to the preimplementation phase (ending in 2020).

### Intervention

The CQFD-I program includes 5 strategies rooted in universal, selective, and indicated interventions. We will provide more detailed information on context-specific activities and implementation strategies when reporting the study’s results.

The first strategy (universal) is the implementation of a French-language web portal designed and constructed specifically for the CQFD-I program, accessible to the general population [29]. This strategy pertains to promoting public awareness and education, an approach to suicide prevention for which evidence is still scarce at the general population level [8]. The portal aims to increase literacy regarding symptoms of depression and signs of social isolation, raise awareness of the importance of consulting health care professionals, and facilitate help-seeking by sharing contact information on locally available resources. The portal includes written text and short videos (approximately 5 minutes) organized into thematic sections, the form and content of which were designed collaboratively to ensure cultural appropriateness in both countries. Furthermore, the portal provides information on locally available resources for help-seeking when users indicate that they are located in either of the implementation sites. Decisions about the most appropriate strategies to advertise the web portal to the population were left to the purview of local program implementers.

The second strategy (selective) is the promotion of a web-based training program on depression targeting primary care physicians. This strategy is supported by considerable evidence indicating that educating nonpsychiatric physicians and primary care nurses in detecting and treating depression may lead to measurable impacts on suicide rates at the population level [3]. The training program used for CQFD-I aims to improve physicians’ skills in early identification and establishing the diagnosis of depressive disorders, assessing the risk of suicide, and determining the appropriate care and services for their patients. Participants can engage with the content of the prerecorded training program at their own pace. Its original version was developed in France by a team led by one of the researchers (MW) several years before its inclusion in the multimodal program and improved following feedback from previous users. One of the researchers (JDC) and a local family physician reviewed the program and requested some modifications to ensure its appropriateness for medical practice in Quebec. While the original version is disseminated in France as a 5-hour activity, adaptation resulted in approximately 1 hour of shared content with some skippable sections replaced by Quebec-specific complementary material. Primary care nurses may also participate in the training program as implemented in Quebec.

The third strategy (selective) is the dissemination of suicidal crisis intervention training targeting social workers and other actors who may have to intervene with at-risk populations. Suicidal crisis intervention training is not based on direct evidence of suicide reduction; however, it constitutes a system-level education initiative targeting people at risk for suicidal crisis [3]. This strategy involves a training program designed by one of the researchers (MS) and disseminated using a train-the-trainer model. Structured as a 2-day workshop, this program covers (1) the epidemiology of suicidal behaviors; (2) current etiological models of suicide; (3) evaluation of the risk, urgency, and danger posed by potential suicidal behaviors; (4) crisis intervention techniques; and (5) safety planning. While the workshop has previously been disseminated to health care workers, notably nurses, it is being extended to social workers for the first time in France in the context of this program. The version of this strategy implemented in Quebec includes the same workshop; however, program implementers also included other training programs targeting suicide prevention recognized by the provincial government.

The fourth strategy (indicated) is the standardization of postcrisis outreach, that is, systematically contacting and providing follow-up for people who have used ambulatory crisis intervention services after calling a helpline or when referred by a third party (eg, friends or family, first responders, health care professionals, or other actors in the community). Evidence supporting this strategy indicates that outreach following suicidal ideation crises may prevent suicidal behaviors [3]. In Brest (France), this strategy leverages the recently implemented VigilanS program designed to provide brief contact interventions to people who attempted suicide [30]. In addition to the usual services of the VigilanS program, additional resources were allocated specifically for postcrisis outreach. In Outaouais (Quebec), this strategy involves adapting and modeling care pathways (in Quebec French: *trajectoires de soins*), a foundation of the management model used in the public health and social services system of the province [31].

The fifth strategy (indicated) is the standardization of outpatient monitoring practices for individuals who have sought medical help after a suicide attempt or who are otherwise deemed acutely at high risk of suicide, based on clinical judgment (eg, a suicidal plan involving accessible means). Despite the lack of clear differentiation between outpatient monitoring and postcrisis outreach in the scientific literature [3], these interventions were conceptualized as separate strategies, considering that the target populations are identifiably different and that outpatient monitoring is more intensive. The standardization of outpatient monitoring in France is provided either by the treating general practitioner or psychiatrist (in private or public practice), or by nurse practitioners, according to a decisional algorithm. This algorithm establishes guidelines for the expected intensity and provider of outpatient monitoring, based on whether the patient has previously attempted suicide and whether the current suicidal crisis is psychiatric or psychosocial in nature. In Quebec, similar to postcrisis outreach, the adaptation and modeling of care pathways were used to standardize outpatient monitoring.

### Outaouais Region and Control Sites

Outaouais is one of Quebec’s 18 health regions (in French: *régions sociosanitaires*), which are administrative divisions used for planning, organizing, and delivering health and social services for the residents of a geographic area. Tables 1 and 2 summarize key characteristics of Outaouais and the 14 other health regions with a population of at least 50,000 individuals that could be included as control sites. We present the most recent data at the beginning of the program’s implementation (before September 1, 2020).

**Table 1:** Key sociodemographic characteristics of the 15 health regions in Quebec with a population of at least 50,000 individuals [32-35].

Health region^a^	Population in 2019, n	Population aged ≥65 years in 2019, n (%)	Population density/km^2^, 2019	Living in materially deprived areas in the 2016 census (%)^b^	Living in socially deprived areas in the 2016 census (%)^b^
Bas-Saint-Laurent	197,530	51,153 (25.9)	8.9	58.74	19.16
Saguenay–Lac-Saint-Jean	278,032	63,589 (22.87)	2.8	49.19	27.72
Capitale-Nationale	751,442	159,903 (21.28)	40.4	17.49	42.63
Mauricie et Centre-du-Québec	520,711	123,564 (23.73)	12.3	55.60	33.25
Estrie	489,986	108,984 (22.24)	38.5	43.70	38.71
Montréal	2,066,035	337,071 (16.31)	4140.4	42.86	63.46
*Outaouais^c^*	*397,007*	*65,561 (16.51)*	*13.0*	*37.74*	*42.62*
Abitibi-Témiscamingue	147,634	28,448 (19.27)	2.6	55.94	28.15
Côte-Nord	90,717	17,767 (19.59)	0.3	61.44	15.80
Gaspésie-Îles-de-la-Madeleine	90,456	24,933 (27.56)	4.5	82.78	11.85
Chaudière-Appalaches	428,969	94,012 (21.92)	28.5	35.97	23.38
Laval	439,583	77,532 (17.64)	1786.9	33.91	30.21
Lanaudière	515,727	95,694 (18.56)	41.9	47.00	25.87
Laurentides	620,648	117,429 (18.92)	30.2	37.24	36.20
Montérégie	1,423,109	266,729 (18.74)	71.4	31.26	33.78

^a^The regions are ordered by official region numbers.

^b^Proportion of the regional population living in dissemination areas that ranked in the bottom 40% (quintiles 4 to 5) of the province for material or social deprivation indices in the 2016 census. Absolute values not included to avoid confusion with 2019 data.

^c^Italics indicate the implementation site.

**Table 2:** Key suicide-related characteristics of Quebec’s 15 health regions with a population of at least 50,000 individuals [32-35].

Health region^a^	Primary care physician ratio per 1000, 2019 to 2020^b^	Suicide rate per 100,000 (age-adjusted^c^), average of the years 2016 to 2019	Male suicide rate per 100,000 (age-adjusted^c^), average of the years 2016 to 2019	Female suicide rate per 100,000 (age-adjusted^c^), average of the years 2016 to 2019	ER^d^ visits for suicide attempt rate per 100,000 (age-adjusted^c^), average of the years 2016 to 2019
Bas-Saint-Laurent	0.85	14.1	22.6	5.7	27.4
Saguenay–Lac-Saint-Jean	0.82	15.7	25.5	5.6	36.7
Capitale-Nationale	0.83	13.7	20.7	6.7	75.1
Mauricie et Centre-du-Québec	0.73	15.1	22.6	7.5	12.9
Estrie	0.78	14.4	21.9	7.0	56.9
Montréal	0.76	10.0	14.3	5.9	42.3
*Outaouais^e^*	*0.72*	*15.5*	*24.9*	*6.4*	*46.8*
Abitibi-Témiscamingue	0.78	21.8	29.2	14.3	63.8
Côte-Nord	0.73	17.4	26.7	7.9	81.5
Gaspésie-Îles-de-la-Madeleine	1.14	19.0	29.3	8.7	33.7
Chaudière-Appalaches	0.71	17.2	25.3	8.8	31.0
Laval	0.71	7.8	12.6	3.3	46.5
Lanaudière	0.65	13.1	22.2	4.0	30.9
Laurentides	0.65	15.2	23.9	6.5	34.8
Montérégie	0.63	11.7	17.6	5.9	102.7

^a^The regions are ordered by official region numbers.

^b^The financial year starts on April 1 and ends on March 31.

^c^Due to discrepancies between data sources, population structure for age adjustment uses 2011 data for suicide rates and 2016 data for emergency room visits. We will ensure a uniform age adjustment methodology when reporting study results.

^d^ER: emergency room.

^e^Italics indicate the implementation site.

With a population of 397,007 in 2019, Outaouais was Quebec’s 10th most populous health region, with the second lowest proportion of residents aged 65 years or more at 16.5% (n=65,561) [32]. Its population density was the seventh lowest [33]. To compare regions’ economic and social profiles, we used the 2016 Canadian material and social deprivation indices, which are indicators compiled at the neighborhood level after each national census [36,37]. Material deprivation is based on median income, educational attainment, and employment, whereas social deprivation accounts for the proportion of people living alone; those who are separated, divorced, or widowed; and single-parent households [36]. In 2016, 37.74% (139,740/370,300) of the population of Outaouais lived in more materially deprived neighborhoods (sixth rank), and 42.62% (157,835/370,300) lived in more socially deprived neighborhoods (14th rank) [34].

The public health and social services of Outaouais have been under the jurisdiction of a single regional authority since 2015 [38]. This is also the case for most regions included in Tables 1 and 2, except for the following: Capitale-Nationale (n=2 distinct health authorities), Montréal (n=10), Côte-Nord (n=2), Gaspésie-Îles-de-la-Madeleine (n=2), and Montérégie (n=3). In the 2019 to 2020 financial year, 437 general practitioners billed universal health insurance of Quebec for services provided in Outaouais, with two-thirds using primary care–specific codes (Données de facturation RAMQ des médecins omnipraticiens, unpublished data, December 2023). This corresponds to 0.72 primary care physicians per 1000 residents (sixth rank). From 2016 to 2019, age-adjusted suicide rate of Outaouais averaged 15.5 per 100,000 overall (11th rank), 24.9 per 100,000 among male individuals (11th rank), and 6.4 per 100,000 among female individuals (seventh rank) (l’Infocentre de santé publique, unpublished data, November 2023). The average rate of hospitalizations for suicide attempts in Outaouais was 53.4 per 100,000 from 2016 to 2019 (eighth rank), and the average rate of ER visits for suicide attempts was 46.8 per 100,000 (10th rank) [35].

### Brest Métropole and Control Sites

Brest Métropole, located in the Finistère department and the Brittany region, is one of the 21 French metropolises, geographically defined entities described as public institutions for intermunicipal cooperation comprising varying numbers of individual communes [39,40]. Excluding areas that have been given a special status under French law, Brest Métropole is one of the 19 metropolises with a similar status, the others of which will serve as control sites [39]. Given that we are currently working with a national public health agency of France, Santé publique France, to extract data at the metropolis level, we could not include it in table form for this paper. In 2019, the population of Brest Métropole was 211,156, making it the least populated among French metropolises [40,41]. The 8 communes of Brest Métropole also represented the lowest number of constituent municipalities compared to other metropolises, which ranged from 12 to 90 [40]. As of January 1, 2020, based on 2019 population and territorial data, Brest Métropole ranked 11th out of 19 for the proportion of inhabitants aged 65 years or more at 18.3% and ninth for population density with 967 inhabitants per square kilometer [42].

### Outcomes and Data Sources

Primary outcomes, which include the suicide mortality rates, hospitalization rates for suicide attempts, and ER visit rates for suicide attempts, will be obtained monthly across 3 phases: preimplementation (September 2015 to August 2020), implementation (September 2020 to August 2022), and postimplementation (September 2022 to August 2024) phases. Data will be collected for both the implementation and control sites in both countries.

We will access Quebec data from the Integrated Chronic Disease Surveillance System database available at the Institut national de santé publique du Québec (INSPQ) [43]. In Quebec, suicide deaths are recorded in the Registre des évènements démographiques–Fichier des décès database and correspond to the *International Classification of Diseases, 10th Revision* (*ICD-10*) codes X60 to X84 and Y87.0 [35]. Data on hospitalizations for suicide attempts are recorded in the Maintenance et exploitation des données pour l’étude de la clientèle hospitalière system and correspond to *ICD-10* codes X60 to X84 and Y87.0. We will only consider hospitalization cases when acute care is the documented reason for admission [35]. ER visits for suicide attempts are recorded in the Banque de données communes des urgences, which includes suicide attempts as one of the options that may be selected as the visits’ main reason by the nursing staff during triage [35]. For rate calculations, we will use the regional population per calendar year from official data published by the Ministère de la Santé et des Services sociaux du Québec [32]. We will benefit from technical support through collaboration with the INSPQ to ensure access to the most appropriate data sources and validate our data extraction procedures.

We will access France data through the Direction de la recherche, des études et des statistiques, a branch of the French Ministry of Health managing the national health data system (*système national de données de santé*) [44]. In France, suicide deaths are recorded by the Centre d’épidémiologie sur les causes médicales de décès and correspond to *ICD-10* codes X60 to X84 [45]. Data on hospitalizations for suicide attempts are available from the Programme de Médicalisation des Systèmes d’Information and correspond to *ICD-10* codes X60 to X84 [45]. ER visits for suicide attempts are available in the Oscour database of ER data and correspond to *ICD-10* codes X60 to X84, excluding X65 (self-intoxication with alcohol), but including other intoxication-related codes selected by a panel of French experts [45]. For rate calculations, we will use metropolis-level data for each calendar year from the Institut national de la statistique et des études économiques. We will collaborate with Santé publique France for technical support to ensure access to the most appropriate data sources and to validate our data extraction procedures.

### Control Sites Selection

Including control sites enables comparison to a counterfactual scenario where the context is similar in all respects except for the CQFD-I strategies. This approach reflects the hypothetical outcomes if the program had not been implemented [24]. Considering that our main hypothesis does not depend on the characteristics of control sites, we will pool all sites within a single control group for the primary analyses. However, we also hypothesized that study results will be sensitive to control site selection. Therefore, we will conduct sensitivity analyses informed by area characteristics expected to influence population-level suicidal behaviors throughout the program implementation. Some relevant area-level data for’ the health regions of Quebec are presented in Tables 1 and 2, and similar indicators are being extracted for French metropolises. Several of these indicators have been used as matching variables in previous studies cited in the Introduction section, that is, population size, population density or remoteness, relative socioeconomic disadvantage, general physicians’ availability, and past suicide rates [19-21]. Given that some suicide prevention programs have found differences based on age groups and sex, we also considered the proportion of the population aged 65 years or older and sex-specific suicide rates. We included the rates of ER visits for suicide attempts as a potential indication of area-specific differences in service use that would interact with the CQFD-I program’s strategies.

### Statistical Methods

We will use the software Stata (StataCorp LLC) to analyze data with a segmented time series model in 3 phases (preimplementation, implementation, and postimplementation phases) and 2 arms (intervention and control areas), conforming to the following equation [27]: Y_t_ = β_0_ + β_1_T_t_ + β_2_X_i_ + β_3_X_p_ + β_4_(T_t_ – t_i_)X_i_ + β_5_(T_t_ – t_p_)X_p_ + β_6_G + β_7_GT_t_ + β_8_GX_i_ + β_9_GX_p_ + β_10_G(T_t_ – t_i_)X_i_ + β_11_G(T_t_ – t_p_)X_p_ + ε_tG_. In this equation, T_t_ is the time elapsed since the beginning of the preimplementation period (September 1, 2015); X_i_ and X_p_ are dummy variables, respectively representing the implementation and postimplementation phases (X=1 during this phase and X=0 during other phases); t_i_ and t_p_ are the first time points of the implementation and postimplementation phases; G=1 for cases and G=0 for controls; and ε_tG_ represents random error that combines ε_t1_ for cases and ε_t0_ for controls.

For all 3 outcome variables (suicide mortality, hospitalizations for suicide attempts, and ER visits for suicide attempts), we will generate monthly rates for each of the cases and control areas throughout the study period (September 2015 to August 2024, n=120 months). For our main analyses, we will adjust monthly rates by age and sex using direct standardization based on yearly data, with the year 2019 serving as the reference population for each implementation site. We will fit the above model using ordinary least square regression, with β_0_ through β_5_ included as pooled values between control areas. We will test for autocorrelation up to 12 lags to account for potential seasonality in our monthly data and adjust the model as per the results. In case of seasonality effect, we will also attempt using Fourier terms or flexible spline functions to fit the data better [46]. In our main analysis for each implementation site, we will treat the implementation phase as a transition period [47] and isolate the program-specific effects: (1) change in rates between the preimplementation and the postimplementation phases (β_8_ + β_9_), and (2) change in trend between the preimplementation and the postimplementation phases (β_10_ + β_11_).

For sensitivity analysis, we will replicate the calculations described in this section with various combinations of control areas pooled within the control group. These exploratory analyses will consider various cutoffs for each of the indicators presented in Tables 1 and 2 as potential criteria for control selection. In addition, we will use any information available at the time of data analysis to identify other suicide prevention programs or services that may have differentially impacted the implementation sites or specific control areas, and to justify testing other combinations of control groups. Furthermore, we will test alternative time series models, including 2 phases instead of 3, using bimonthly and trimonthly rather than monthly data, and stratifying by sex and age groups when allowed by power considerations. We will clearly report these analyses as exploratory to avoid distracting from the main study results.

### Statistical Power

For the Outaouais region (n=397,007), the statistical power to detect a 20% decrease from the 2016 to 2019 averages at α=.05 would be 33.1% for suicide mortality, 85.7% for hospitalizations, and 80.4% for ER visits [48]. We do not yet have access to suicidal behavior data specifically for Brest Métropole; however, we estimated the statistical power based on its 2019 population (n=211,156) and published data about the Finistère department as a whole: the suicide rate was 23.4 per 100,000 in 2015 and the hospitalization rate was 227.8 per 100,000 in 2017 [45]. From this lower population but considerably higher rates of suicidal behaviors, the statistical power to detect a 20% decrease at α=.05 in Brest Métropole would be 26.8% for suicide mortality and 99.7% for hospitalizations. We did not find a reported rate of ER visits for suicide attempts in Finistère for the same period; however, cursory calculations indicate a base rate approximating 130 per 100,000 and a statistical power higher than 90%.

Given the low incidence of suicide in the general population, statistical power is often an issue when conducting pre-post rate comparisons. This has been reported as a justification to aggregate suicide mortality and suicide attempts in OSPI-Europe [12]. However, the statistical power benefits of variables aggregation would be marginal compared to including hospitalizations and ER visit rates as distinct primary outcomes, both of which can be assessed with satisfactory statistical power. Moreover, interrupted time series will provide additional benefits relating to the statistical power of this study in two important ways: (1) including a high number of repeated observations (n=120 months) [49] and (2) assessing the intervention effect on the outcome slope in addition to their level [50]. Considering the very different implications of death rates and indicators influenced by service use, we will analyze each primary outcome separately.

### Ethical Considerations

Evaluation of the *CQFD-I* program was approved by the research ethics board of the Centre intégré de santé et de services sociaux de l'Outaouais in May 2021 (2020-305_171) and by the Comité d’éthique de Brest in March 2023 (B2023CE.18). No consent to participate is required as we will exclusively interact with nonidentifying data in the context of official procedures overseen by governmental bodies in both implementation jurisdictions. We will only extract and report data aggregated at the population level. This outcome evaluation will thus constitute a minimal-risk project for all individuals involved, with no risk to the privacy or confidentiality of any participant. Additional precautions will be undertaken as required by the public agencies providing access to data in Quebec and France.

## Results

The CQFD-I program was conceptualized and refined from 2014 to 2020 through a series of meetings and negotiations between the 2 international partners, culminating in a version suited for implementation in Quebec and France despite differences in their medical and psychosocial service structures. The CQFD-I program’s timeline includes 3 phases: preimplementation (2015 to 2020), implementation (2020 to 2022), and postimplementation (2022 to 2024) phases. The implementation phase commenced on September 1, 2020, marked by the initial official meetings held within that month to brief stakeholders on the program’s strategies. Considering that (1) Brest Métropole was constituted in January 2015 [39] and (2) a law initiating a major health and social services reform was passed in Quebec in February 2015 [51], we define September 1, 2015, as the beginning of the preimplementation phase of the study. This 5-year period for data collection was established to ensure a sufficiently long baseline to reflect changes in policy. All 5 strategies of the CQFD-I program were fully implemented by September 1, 2022, marking the beginning of the postimplementation phase. For evaluation purposes, August 31, 2024, will be the last day of the postimplementation phase.

This project is funded by the Ministère de la Santé et des Services Sociaux du Québec, with access to data and expertise representing in-kind contributions from Santé publique France and INSPQ. Under the responsibility of the first author, data will be collected from public health databases in September 2027 to account for the delays necessary to confirm suicide deaths in official registers. Data analysis will be performed from September 2027 to June 2028.

## Discussion

### Anticipated Findings

In this paper, we present the study protocol for the outcome evaluation of the CQFD-I program, a multimodal suicide prevention program currently implemented in 2 sites, that is, Brest Métropole in Brittany, France, and the Outaouais region in Quebec, Canada. We hypothesize that after 2 years of the CQFD-I program’s implementation, there will be a 20% reduction in suicide mortality, hospitalizations due to suicide attempts, and ER visits for suicide attempts compared to control sites. Through this study, we will generate data for the first time on the effects of implementing a multimodal suicide prevention program at the population level in 2 countries not exclusively located in Europe. In addition to its general contribution to currently limited knowledge about the effects of multimodal suicide prevention programs, this study represents a proof of concept of sharing international resources to design a program targeting populations speaking a common language but residing in very different jurisdictions. Accordingly, positive results from this study would have profound implications on the potential to leverage international partnerships to disseminate best practices in suicide prevention, a promising avenue for low-income countries and underserved linguistic minority groups.

One limitation of this protocol is the reliance on administrative databases to ascertain suicide-related outcomes. While this allows for long-term follow-up across implementation phases and ensures comparable data quality between implementation and control sites, it may limit the interpretability of results. For instance, Quebec includes *ICD-10* code Y87.0 (sequelae of intentional self-harm, assault, and events of undetermined intent) for suicide attempts, whereas French databases do not. Even within jurisdictions, definitions differ: in Quebec, suicide attempt hospitalizations are coded with *ICD-10*, while ER visits rely on triage-based clinical judgment. To address these discrepancies, we will avoid assuming data source equivalence. First, suicide attempt rates will only be compared within the same jurisdiction. Second, each outcome—suicide mortality, hospitalizations, and ER visits—will be analyzed separately rather than combined for statistical power. While we expect all outcomes to decrease if the CQFD-I program is effective, reductions in only some outcomes would still be valuable results and warrant further research.

### Conclusions

Results may guide future research by offering valuable insights into directing suicide prevention efforts for geographically defined populations. The CQFD-I program created opportunities for participants from 2 distinct jurisdictions to reflect on the unique aspects of the health and social services systems and challenge assumptions that might otherwise limit their ability to innovate in suicide prevention. Program development and implementation benefited from sharing expertise and resources; however, they also highlighted new challenges for researchers to address regarding the operationalization and integration of suicide prevention strategies at the subnational level. Each of the CQFD-I program’s components had to be designed and operationalized to accommodate implementation in 2 distinct jurisdictions, a task that involved both anticipated and unforeseen challenges. The web portal required tailored promotional strategies aligned with local information consumption habits, demanding creativity from program implementers. Dissemination of the depression training program had to meet the high continuing education standards of primary care physicians, while suicide assessment and management training needed to be adapted to the staff training approaches of existing organizations. Standardizing postcrisis outreach and outpatient monitoring required a thorough understanding of clinical and managerial practices across multiple disciplines and institutions. The program’s implementation will be the topic of an upcoming paper that will go hand in hand with the results of the study described in this protocol.

## Abbreviations

CQFD-I: Coopération Québec-France contre la Dépression et l’isolement
ER: emergency room
ICD-10: International Classification of Diseases, 10th Revision
INSPQ: Institut national de santé publique du Québec
OSPI-Europe: Optimizing suicide prevention programs and their implementation in Europe
TREND: Transparent Reporting of Evaluations with Nonrandomized Designs

## Data Availability

The datasets analyzed during this study will be available as permitted and recommended by the public health organizations that will provide access to these data, that is, the Institut national de la santé publique du Québec and Santé publique France.
